# Case Report: Qizhi Yishen Capsule-induced bone marrow granulomas and severe myelosuppression

**DOI:** 10.3389/falgy.2026.1889878

**Published:** 2026-07-08

**Authors:** Ping Xie, Yuting Zhang, Jinli Jiang, Caiping Chen, Yingying Wang, Liangming Liu

**Affiliations:** 1 Department of Infectious Diseases, Songjiang Hospital Affiliated to Shanghai Jiao Tong University School of Medicine, Shanghai, China; 2 Department of Infectious Diseases, Shanghai Songjiang Clinical Medical College, Nanjing Medical University, Shanghai, China

**Keywords:** adverse drug reaction, bone marrow granulomas, case report, myelosuppression, Qizhi Yishen Capsule, sinomenine

## Abstract

**Background:**

Drug-induced bone marrow injury is an under-recognised adverse drug reaction (ADR) that can present with fever, cytopenias, and eosinophilia. Drug-induced bone marrow granuloma formation remains exceedingly rare among ADRs, and to our knowledge, no such cases associated with traditional Chinese medicine (TCM) formulations have been previously reported.

**Case presentation:**

A 79-year-old woman with chronic kidney disease (CKD) developed fever and marked eosinophilia after six weeks of treatment with Qizhi Yishen Capsule, a sinomenine-containing TCM formulation. She rapidly progressed to agranulocytosis and pancytopenia. Bone marrow biopsy revealed non-caseating granulomas with severe myelosuppression and grade 3 fibrosis. Immunohistochemistry showed CD3⁺ T-cell predominance within granulomas with CD20⁺ B cells absent. The Naranjo score was 7 (probable ADR). Treatment with glucocorticoids and granulocyte colony-stimulating factor (G-CSF) resulted in complete hematologic recovery.

**Conclusion:**

This case documents bone marrow granulomas and severe myelosuppression following Qizhi Yishen Capsule administration, a sinomenine-containing TCM formulation. A Naranjo score of 7 (probable) and immunohistochemical evidence of CD3⁺ T-cell infiltration within the granulomata support a drug-related, cell-mediated pathogenesis. Clinicians should consider drug-induced bone marrow toxicity in patients receiving this formulation, particularly those with CKD.

## 1 Introduction

Adverse drug reactions (ADRs) encompass a broad clinical spectrum, ranging from mild laboratory abnormalities to life-threatening systemic conditions (1). Among the most diagnostically challenging are ADRs that produce isolated bone marrow injury, as they often lack pathognomonic features and overlap substantially with infectious, autoimmune, or malignant aetiologies (2). Drug-induced haematological toxicity, including agranulocytosis and pancytopenia, represents a potentially fatal yet under-recognised form of ADR; when fever, eosinophilia, and cytopenias co-occur following drug exposure, systematic causality assessment is essential to distinguish drug-induced injury from competing diagnoses.

Traditional Chinese medicine (TCM) formulations are increasingly recognized as potential triggers of severe ADRs. Their complex, multi-component compositions complicate the identification of specific toxic components, and the full spectrum of haematological toxicity associated with TCM remains incompletely characterised (3).

Qizhi Yishen Capsule, widely used as adjunctive therapy for chronic kidney disease (CKD), contains *Sinomenium acutum* (Qingfengteng), from which sinomenine — an alkaloid previously reported to cause agranulocytosis (4, 5) — is derived. Patients with CKD may be at heightened risk due to impaired sinomenine elimination. However, bone marrow granuloma formation associated with sinomenine-containing preparations has not been previously described.

In this article, we report a case of bone marrow granulomas and severe myelosuppression following Qizhi Yishen Capsule administration (Naranjo score = 7, “probable”), with T-cell- predominant granulomatous inflammation documented by immunohistochemistry.

## 2 Case description

### 2.1 Presentation and admission

A 79-year-old woman was admitted to the hospital, on November 9, 2025, for evaluation of fever of 6 days’ duration. The fever peaked at 39 °C and was accompanied by chills, generalized fatigue, and anorexia. She denied cough, dyspnea, abdominal pain, dysuria, or skin lesions during the illness. Her past medical history included hypertension (10 years, well controlled with losartan-hydrochlorothiazide), cholecystolithiasis, and CKD (2 years). She had no known drug allergies.

On admission, vital signs showed a temperature of 38.5 °C, with normal heart rate and blood pressure. Physical examination was unremarkable, with no lymphadenopathy, no rash, and normal cardiopulmonary and abdominal findings. Initial laboratory studies revealed thrombocytopenia (PLT 78 × 10⁹/L), eosinophilia (20.6%, absolute count 1.28 × 10⁹/L), elevated C-reactive protein (CRP 111.88 mg/L), and procalcitonin (PCT 1.82 ng/mL). Cytokine levels were markedly elevated: serum soluble interleukin-2 receptor (sIL-2R) at 2461.00 U/mL (reference: 228–724 U/mL), interleukin-6 (IL-6) at 21.93 pg/mL (reference:0–7 pg/mL), interleukin-10(IL-10) at 18.66 pg/mL (reference:0–9.5 pg/mL), and ferritin at 449.07 μg/L (reference:0–160 μg/L). Liver function and cardiac enzymes were normal, while renal function was mildly impaired. Non-contrast chest CT showed changes consistent with chronic bronchitis.

### 2.2 Drug exposure history

A detailed drug history obtained on hospital day 5 revealed that the patient had been taking Qizhi Yishen Capsule (major components: Astragalus membranaceus, Rehmannia glutinosa, Ligustrum lucidum, Hirudo nipponia, stir-fried Bombyx batryticatus, Eupolyphaga sinensis, prepared Rheum officinale, Gymnema sylvestre, Sinomenium acutum, and Plantago) for 6 weeks prior to the onset of fever as an adjunctive treatment for CKD. She had discontinued the capsules on her own initiative due to decreased appetite before admission. Losartan-hydrochlorothiazide had been taken continuously for 10 years without prior adverse effects.

### 2.3 Hospital course

Initial empiric antibiotic therapy with ceftazidime2.0 g intravenously (IV) every 12 h was initiated for suspected infectious fever. Despite 4 days of antibiotic therapy, fever persisted and hematologic parameters deteriorated rapidly: white blood cell (WBC) count fell to 1.89 × 10⁹/L with an absolute neutrophil count (ANC) of only 0.03 × 10⁹/L, while the eosinophil fraction rose further to 37.61% (absolute count 0.71 × 10⁹/L). Cytokine levels remained markedly elevated: sIL-2R 4214 U/mL, and ferritin 617.16 μg/L. Inflammatory markers partially declined: PCT 0.38 ng/mL, CRP 63.00 mg/L.

Infectious workup including blood cultures, fungal biomarkers, serologies for Epstein–Barr virus (EBV), cytomegalovirus (CMV), human immunodeficiency virus (HIV), interferon gamma release assay (IGRA), and parasitic infections were negative. Nasopharyngeal swab targeted next-generation sequencing (tNGS) revealed no clinically significant pathogens.

Hematology consultation was obtained on hospital day 5. The working diagnosis was shifted from infectious to non-infectious etiology. Bone marrow aspiration and biopsy were performed on day 6. Treatment was initiated with methylprednisolone 40 mg/day IV and recombinant granulocyte colony-stimulating factor (G-CSF) 150 µg/day subcutaneously. However, the patient remained febrile and ANC continued to decline, reaching a nadir of 0.01 × 10⁹/L on days 6–9.

Based on the exclusion of infectious, autoimmune, and malignant aetiologies, the presentation was attributed to Qizhi Yishen Capsule-induced haematological toxicity. Methylprednisolone was replaced with dexamethasone 5 mg/day IV (day 12). The patient became afebrile within 48 h, and blood counts began to recover. She was discharged on hospital day 18.

## 3 Timeline

## 4 Diagnostic evaluation, treatment, and follow-up

### 4.1 Laboratory and ancillary investigations

Immunologic studies revealed markedly elevated IgE (1007.04 IU/mL, reference: 0–100 IU/mL), sIL-2R peaking at 4,214 U/mL, and persistently elevated IL-6 and IL-10, indicating systemic T-cell activation. Antinuclear antibodies, tumor markers, and T-lymphocyte counts were within normal limits. No abnormal lymphocytes seen on peripheral blood smear. Echocardiography and cervical, axillary, and inguinal lymph nodes ultrasound showed no abnormalities (Table 1).

**Table 1:** Timeline.

Time point	WBC ( × 10⁹/L)	Hb (g/L)	PLT ( × 10⁹/L)	ANC ( × 10⁹/L)	Eos ( × 10⁹/L)	Key events
23 Sep 2025	–	–	–	–	–	Qizhi Yishen Capsule initiated for CKD
4 Nov 2025	–	–	–	–	–	Fever (Tmax39 °C); capsule self-discontinued
9 Nov 2025 (Day 1)	6.22	123	78	2.52	1.28	Admission. Eos 20.6%. Ceftazidime started
13-14 Nov 2025 (Day 5-6)	1.89	119	128	0.03	0.71	Agranulocytosis. Hematology consult → ceftazidime stopped; methylprednisolone + G-CSF started; bone marrow biopsy performed
17 Nov 2025 (Day 9)	1.94	101	120	0.01	0.06	Persistent fever, agranulocytosis
20 Nov 2025 (Day 12)	2.98	106	164	0.48	0.07	Biopsy: hypocellularity (∼10%), MF-3 fibrosis, non-caseating granulomata with CD3⁺ T cells. Drug-induced bone marrow injury suspected. Switched to dexamethasone
22 Nov 2025 (Day 14)	30.4	104	161	22.39	0.04	Fever resolved. WBC rebound
26 Nov 2025 (Day 18)	16.8	100	117	12.56	0.03	Dexamethasone discontinued; discharge
24 Dec 2025 (1-month follow up)	8.5	118	127	5.3	0.04	CBC normalized. Repeat bone marrow biopsy declined
23 Mar 2026 (4-month follow up)	5.32	121	131	2.88	0.08	Sustained remission

WBC, white blood cell count; Hb, hemoglobin; PLT, platelet count; ANC, absolute neutrophil count; CBC, complete blood count; Eos, absolute eosinophil count; –, not tested; G-CSF, granulocyte colony-stimulating factor; CKD, chronic kidney disease.

**Figure 1:**
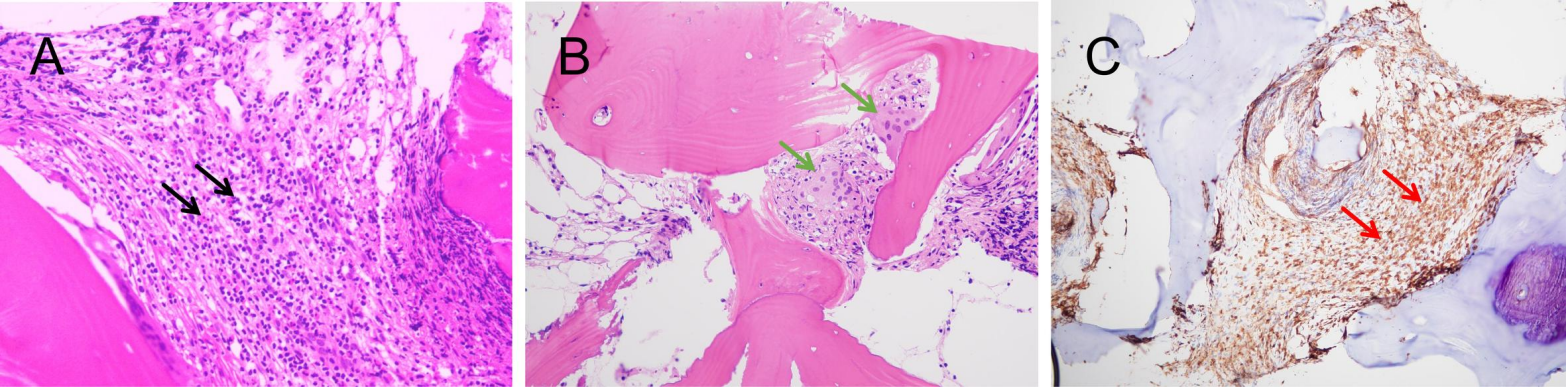
Bone marrow biopsy histopathology. **(A)** Prominent eosinophilic infiltration. **(B)** Non-caseating granuloma with multinucleated giant cells. **(C)** Intra-granulomatous CD3⁺ T-cell predominance with absent CD20⁺ B cells.

Bone marrow cytology showed hypocellularity with no excess blasts, no increase in non-hematopoietic cells, and a differential dominated by segmented neutrophils and eosinophils. Bone marrow biopsy demonstrated marked hypocellularity, with approximately 10% nucleated cells and severely reduced trilineage hematopoiesis. Reticulin staining revealed grade 3 myelofibrosis (MF-3). Immunohistochemistry showed prominent eosinophilic infiltration (Figure 1A) and granulomatous nodules composed of multinucleated giant cells without caseation (Figure 1B). Intra-granulomatous lymphocytes were predominantly CD3⁺ T cells; CD20⁺ B cells were absent (Figure 1C). Fluorescence *in situ* hybridization (FISH) showed no PDGFRA/PDGFRB rearrangement; conventional cytogenetics was normal (46,XX); *BCR*::*ABL1* was negative. Flow cytometry revealed lymphocytes 54.39% and eosinophils 20.02%, with no other abnormalities. Bone marrow acid-fast staining and bacterial culture were negative.

### 4.2 Causality assessment

Causality between Qizhi Yishen Capsule and the observed bone marrow injury was assessed using the Naranjo scale. The total score was 7, indicating a “probable” ADR (6). The detailed Naranjo causality assessment is provided in Supplementary Table 9. Contributing items included prior reports of sinomenine-associated agranulocytosis (+1 points) (4, 5), onset following drug administration (+2 points), improvement after discontinuation (+1 points), systematic exclusion of alternative causes (+2 points), and objective laboratory confirmation of pancytopenia by serial complete blood counts (+1 point). Therefore, Qizhi Yishen Capsule was considered the most probable cause of bone marrow injury with T-cell-mediated granulomatous inflammation in this patient.

### 4.3 Differential diagnosis

The following conditions were systematically evaluated and excluded: (1) Infectious causes of bone marrow granuloma (tuberculosis, fungi, brucellosis) — bone marrow acid-fast stain, culture, and tNGS were negative; IGRA was negative. (2) Hematologic malignancies (chronic eosinophilic leukemia, lymphoma) — molecular genetics and bone marrow morphology excluded clonal disorders (PDGFRA/PDGFRB/BCR::ABL1 all negative). (3) Autoimmune diseases (sarcoidosis, connective tissue disease) — antinuclear antibody panel was negative. (4) Drug reaction with eosinophilia and systemic symptoms (DRESS) syndrome — the RegiSCAR scoring system was applied: eosinophilia ≥20% contributed +2 points and systematic exclusion of alternative diagnoses with ≥3 investigations contributed +1 point, offset by a 1-point deduction for the absence of rash (−1 point), yielding a total score of 2 (possible DRESS range) (7) therefore, the diagnostic criteria were not fulfilled. The detailed RegiSCAR scoring assessment is provided in Supplementary Table 10. Losartan-hydrochlorothiazide, which the patient had taken for 10 years, was considered but deemed unlikely because cytopenias resolved despite continued use of this medication throughout treatment and follow-up, further supporting Qizhi Yishen Capsule as the causative agent.

### 4.4 Treatment and follow-up

Key treatment measures included: (1) immediate discontinuation of the suspected offending drug (Qizhi Yishen Capsule had already been self-discontinued before admission); (2) glucocorticoids: methylprednisolone 40 mg/day IV did not achieve satisfactory control of fever; however, switching to dexamethasone 5 mg/day IV resulted in defervescence within 48 h; (3) G-CSF 150 µg/day subcutaneously from day 6 to support neutrophil recovery. At 1-month follow-up, WBC was 8.5 × 10⁹/L, ANC 5.3 × 10⁹/L, eosinophils 3%, Hb 118 g/L, and PLT 127 × 10⁹/L. Repeat bone marrow biopsy was declined by the patient due to advanced age. She remained well at 4-month follow-up.

## 5 Discussion

The present case illustrates severe drug-induced bone marrow injury with granulomatous inflammation attributable to Qizhi Yishen Capsule, characterised by fever, eosinophilia, bone marrow granulomas, and pancytopenia. The Naranjo score of 7 supports probable drug causality, with systematic exclusion of infectious, malignant, and autoimmune causes further substantiating this diagnosis.

Drug-induced bone marrow injury can closely mimic haematological malignancies (8, 9). In our patient, the combination of severe pancytopenia, myelofibrosis (MF-3), granuloma formation, and marked eosinophilia necessitated urgent exclusion of chronic eosinophilic leukaemia, myeloproliferative neoplasms, and lymphoma. *PDGFRA*/*PDGFRB* rearrangement by FISH, *BCR::ABL1* fusion testing, conventional cytogenetics, and bone marrow flow cytometry were all negative, effectively excluding a clonal haematological process. DRESS syndrome was additionally considered given the co-occurrence of fever and eosinophilia. Application of the RegiSCAR scoring system yielded a total score of 2 (possible DRESS range), primarily reflecting the complete absence of rash, lymphadenopathy, and hepatic involvement — all cardinal features of the classic DRESS phenotype (10). The diagnostic criteria for DRESS syndrome were therefore not fulfilled. Although the “DRESS sine rash” variant has been proposed (11), it remains without validated diagnostic criteria. Taken together, the absence of clonal markers, failure to meet established DRESS criteria, and complete haematological recovery with corticosteroid therapy collectively support a diagnosis of bone marrow injury with granulomatous inflammation following Qizhi Yishen Capsule administration.

Mechanistically, the predominance of CD3⁺ T cells within the bone marrow granulomata strongly implicates a T-cell-mediated immune mechanism (12). The drug or its metabolites activate drug-specific T cells that subsequently release a cascade of cytokines — including IL-5, interferon-gamma (IFN-*γ*), and Tumor Necrosis Factor-alpha (TNF-α) — driving eosinophil recruitment, granuloma formation, and inhibition of hematopoietic progenitor proliferation. The markedly elevated serum sIL-2R (peak 4214 U/mL), IL-6, and IL-10 levels corroborated systemic T-cell activation and immune dysregulation. The development of grade 3 myelofibrosis is consistent with secondary fibrosis driven by the release of profibrotic mediators such as TGF-β within a sustained inflammatory marrow microenvironment (13, 14); complete hematological recovery during follow-up further supports its secondary, reversible nature.

Regarding the causative agent, Qizhi Yishen Capsule contains multiple components; sinomenine, derived from *Sinomenium acutum*, is the most plausible culprit. Sinomenine has been previously reported to cause agranulocytosis (4, 5), and the present patient similarly had pre-existing CKD —a finding congruent with the proposed mechanism that renal insufficiency may predispose to sinomenine-associated hematological toxicity. Although losartan potassium/hydrochlorothiazide has rare reports of causing cytopenias (15), this agent had been well tolerated for years and was never discontinued during treatment; bone marrow function gradually recovered with corticosteroid therapy alone. To our knowledge, this case documents,bone marrow granuloma formation attributable to a sinomenine-containing formulation, expanding the recognised spectrum of sinomenine-associated haematological toxicity beyond isolated agranulocytosis. Given the increasing use of sinomenine in TCM preparations, clinicians should consider haematological adverse effects, particularly in patients with CKD.

With respect to management, early recognition and immediate drug discontinuation are paramount. Given the presence of bone marrow granulomatous inflammation with CD3⁺ T-cell infiltration, systemic glucocorticoids were administered to suppress the T-cell-mediated immune injury.In this case, after 6 days of methylprednisolone (40 mg/day) without adequate fever control, the regimen was switched to dexamethasone (5 mg/day), which was followed by rapid defervescence and haematological recovery. Whether this represented a genuine pharmacological advantage of dexamethasone — attributable to its greater anti-inflammatory potency and longer half-life — or simply reflected the cumulative effect of prolonged immunosuppression cannot be determined from a single case. G-CSF was used as an adjunct to accelerate neutrophil recovery during the period of agranulocytosis.

Several limitations should be acknowledged. First, the compound nature of the herbal preparation precluded identification of the specific sensitizing component. Second, no drug provocation test was performed given the patient's advanced age and severe haematological compromise; accordingly, the Naranjo re-exposure item was scored as not applicable. Furthermore, *in vitro* investigations (e.g., lymphocyte transformation tests) were not conducted owing to the lack of established protocols for multi-herbal formulations. Third, the patient declined a post-discharge bone marrow biopsy, so direct histopathological evidence of granuloma resolution is unavailable. Fourth, this is a single-center case report; generalizability awaits validation by additional case series and multicenter studies.

## 6 Conclusion

In conclusion, this case documents bone marrow granuloma formation and severe myelosuppression following administration of Qizhi Yishen Capsule, a sinomenine-containing TCM formulation. A Naranjo score of 7 (probable) and immunohistochemical evidence of CD3⁺ T-cell infiltration within the granulomata support a drug-related, cell-mediated pathogenesis. Clinicians should consider drug-induced haematological toxicity when unexplained fever, eosinophilia, and cytopenias co-occur in patients receiving sinomenine-containing TCM preparations, even in the complete absence of rash. Regular monitoring of peripheral blood counts is advisable during treatment with sinomenine-containing preparations, particularly in patients with CKD.

## 7 Patient perspective

I originally thought that Qizhi Yishen Capsule, being a traditional Chinese medicine, was very safe, so I kept taking it. Unexpectedly, after developing a high fever, my white blood cell count became lower and lower, and even antibiotics did not work; my family and I were very worried. Later, the doctor carefully asked about all the medications I was taking and then realized that it might be caused by this traditional Chinese medicine. After switching to steroid treatment, the fever subsided and my blood counts gradually improved. I am very grateful for the doctor's carefulness. This experience has taught me that traditional Chinese medicines can also have serious side effects. In the future, when I see a doctor, I must tell the doctor about all the Chinese patent medicines or health supplements I am taking, and cannot assume that “pure natural” means “no side effects”.

## 8 Patient consent

Written informed consent was obtained from the patient for publication of this case report and accompanying images.

## Data Availability

The original contributions presented in the study are included in the article/Supplementary Material, further inquiries can be directed to the corresponding author.
