# Supplementary material for: Case Report: Qizhi Yishen Capsule-induced bone marrow granulomas and severe myelosuppression
Source: Front Allergy. 2026 Jul 8;7:1889878. doi: 10.3389/falgy.2026.1889878 (PMC13388561; doi:10.3389/falgy.2026.1889878)
Supplement: Supplementary file 8 [file Datasheet8.pdf]

CARE
case report guidelines

CARE Checklist of information to include when writing a case report

(cc) BY-NC-ND

Topic	Item	Checklist item description	Reported on Line
Title	1	The diagnosis or intervention of primary focus followed by the words "case report"	Page 1, Title
Key Words	2	2 to 5 key words that identify diagnoses or interventions in this case report, including "case report"	line 10-11
Abstract (no references)	3a	Introduction: What is unique about this case and what does it add to the scientific literature?	line 13-16
	3b	Main symptoms and/or important clinical findings	line 17-22
	3c	The main diagnoses, therapeutic interventions, and outcomes	line 23-24
	3d	Conclusion—What is the main "take-away" lesson(s) from this case?	line 25-29
Introduction	4	One or two paragraphs summarizing why this case is unique (**may include references**)	line 30-57
Patient Information	5a	De-identified patient specific information.	line60
	5b	Primary concerns and symptoms of the patient.	line 61
	5c	Medical, family, and psycho-social history including relevant genetic information	line 63-65
	5d	Relevant past interventions with outcomes	Not
Clinical Findings	6	Describe significant physical examination (PE) and important clinical findings.	line 68-74
Timeline	7	Historical and current information from this episode of care organized as a timeline	line 106
Diagnostic Assessment	8a	Diagnostic testing (such as PE, laboratory testing, imaging, surveys).	line 84-105
	8b	Diagnostic challenges (such as access to testing, financial, or cultural)	line 37-41
	8c	Diagnosis (including other diagnoses considered)	line 125-136
	8d	Prognosis (such as staging in oncology) where applicable	line101-105
Therapeutic Intervention	9a	Types of therapeutic intervention (such as pharmacologic, surgical, preventive, self-care)	line148-152
	9b	Administration of therapeutic intervention (such as dosage, strength, duration)	line 85-89,148-152
	9c	Changes in therapeutic intervention (with rationale)	line 101-103
Follow-up and Outcomes	10a	Clinician and patient-assessed outcomes (if available)	line 101-105
	10b	Important follow-up diagnostic and other test results	line 103-105
	10c	Intervention adherence and tolerability (How was this assessed?)	line 101-105
	10d	Adverse and unanticipated events	Not available
Discussion	11a	A scientific discussion of the strengths AND limitations associated with this case report	line 201-205
	11b	Discussion of the relevant medical literature **with references**.	line 165-167
	11c	The scientific rationale for any conclusions (including assessment of possible causes)	line 156-160
	11d	The primary "take-away" lessons of this case report (without references) in a one paragraph conclusion	line 206-221
Patient Perspective	12	The patient should share their perspective in one to two paragraphs on the treatment(s) they received	line 222-232
Informed Consent	13	Did the patient give informed consent? Please provide if requested	Yes ☑ No ☐
