# Supplementary material for: Case Report: Qizhi Yishen Capsule-induced bone marrow granulomas and severe myelosuppression
Source: Front Allergy. 2026 Jul 8;7:1889878. doi: 10.3389/falgy.2026.1889878 (PMC13388561; doi:10.3389/falgy.2026.1889878)
Supplement: Supplementary file 10 [file Datasheet10.pdf]

RegiSCAR DRESS Validation Scoring System

Drug Reaction with Eosinophilia and Systemic Symptoms (DRESS) - English scoring sheet

Clinical / laboratory feature	Scoring criterion	Score	Patient finding / notes
Fever	Temperature >=38.5 deg C: Yes	0	0
	No or unknown	-1	
Enlarged lymph nodes	At least 2 sites, >1 cm: Yes	+1	
	No or unknown	0	0
Atypical lymphocytes	Present	+1	
	Absent or unknown	0	0
Eosinophilia	700-1,499 cells/uL or 10%-19.9%	+1	
	>=1,500 cells/uL or >=20%	+2	+2
	0-699 cells/uL or <10%	0	
Skin rash extent	Rash involving >50% body surface area	+1	
	No or unknown	0	0
Skin rash suggesting DRESS	At least 2 of: facial edema, infiltration, purpura, scaling/desquamation	+1	
	No: does not meet the above morphology criterion	-1	-1
	Unknown	0	
Skin biopsy suggesting DRESS	Compatible or unknown	0	0
	Not compatible	-1	
Internal organ involvement	One internal organ involved	+1	
	Two or more internal organs involved	+2	
	No internal organ involvement	0	0
Resolution / disease course	Clinical symptoms persist for >=15 days	0	0
	Resolution <15 days or unknown	-1	
Evaluation of alternative causes	At least 3 relevant investigations performed and negative	+1	+1
	Not done, insufficient, positive, or unknown	0	
**Total score:** ____2________		**RegiSCAR classification:** ______Possible DRESS__	

Total score	RegiSCAR classification
<2	No case / excluded DRESS
2-3	Possible DRESS
4-5	Probable DRESS
>=6	Definite DRESS

Notes for atypical/no-rash cases: If there is no rash, the item "Skin rash extent >50% body surface area" is usually scored 0, and the morphology item "Skin rash suggesting DRESS" is scored -1 when facial edema, infiltration, purpura, or scaling/desquamation are absent. There is no separate RegiSCAR score specifically for DRESS sine rash, so this limitation should be acknowledged when applying the score.

Reference: Adapted from the RegiSCAR DRESS validation scoring system, as used in Kardaun et al., *British Journal of Dermatology*, 2013, and commonly reproduced in clinical calculators/reviews. This sheet is for academic documentation and does not replace clinical judgment.
